# Diseases of the Blood and Blood-Vessels

**Published:** 1905-01-21

**Authors:** 


### DISEASES OF THE BLOOD AND BLOOD-VESSELS.

**Arterial Sclerosis.**—Savill[1] defines arterial sclerosis as a chronic generalised thickening or degeneration of the arterial walls, in which after death the lumen does not collapse as usual, and the walls are harder and less elastic than normal. Histologically the condition may occur in any one or more of the three coats of an artery. Atheroma is a condition quite distinct from intimal sclerosis ; it is a patchy, fibroplastic infiltration of the intima prone to undergo necrotic changes, and is visible as raised yellow spots on one side of an artery, which can be felt as beaded thickenings during life ; it affects chiefly the aorta and larger arteries of the trunk and brain. On the other hand, sclerosis of the intima is a more or less uniform thickening all round the lumen of an artery without alteration of colour and without any tendency to necrosis ; it gives a vessel a hard smooth feel like whipcord, and it affects arteries of any size, but is more common in medium and smaller vessels. Atheroma is of no serious pathological importance provided the cardio-vascular system is otherwise fairly healthy. In many cases chronic adventitial and intimal sclerosis is secondary both in time and in importance to changes in the media, or to dilatation of the arterial lumen. Sclerosis of the intima may affect a particular territory only, *e.g.* the renal vessels, and not be a general change. The muscular coat of the arteries is, relatively to the other coats, by far the most important, and in the arteries and arterioles forms about half the thickness of the vessel wall; the intima is only a limiting membrane, the adventitia only a supporting structure. The most important constituent of incipient old age is the loss of the regulator function of the arteries in maintaining the blood-pressure relatively constant. Many morbid changes are met with in the muscular coat; thus a slight degree of atrophy may be found in wasting disorders. Hypermyotrophy is of very frequent occurrence, and supervening on this cloudy swelling, granular degeneration, dilatation and necrosis may occur. The consequences of arterial hypermyotrophy, cloudy and granular degeneration—the generalised changes—are primarily disturbances of the dynamics of the circulation, whereas the effects of necrosis, if unaccompanied by compensatory adventitial or intimal changes, lead to rupture and hæmorrhage. They probably exist in a large proportion of cases of cerebral hæmorrhage. Arterial hypermyotrophy is a distinct clinical and pathological condition, characterised in the first stage mainly by postural vertigo, in the second by evident thickness of the arteries, cardiac hypertrophy, and sometimes cerebral and other hæmorrhages, and in the final stages by circulatory and nutritional disturbances. Sometimes, but not necessarily, it is associated with chronic renal disease, which is only one of its many causes. High arterial tension, sometimes intermittent, but usually persistent, existed in all the cases before arterial degeneration or dilatation occurred, and in many cases after this had taken place, and is the main cause of the condition. Thus of 17 cases there was a history of alcoholic excess in seven, of gout in two, of passing gravel or lithates in six, of chronic renal disease in three. Alcohol plays the leading *rôle* in producing the disease. The three conditions apt to be confused with arterial hypermyotrophy are old age, granular kidney, and other forms of arterial degeneration. In treatment, nitroglycerine has a marked effect in the early stages. If one could prevent hypermyotrophy one could prolong life, for it is the first step to senile decay.

[1] Lancet, Sept. 24.

*(To be continued.)*

